# Eggs survive through avian guts—A possible mechanism for transoceanic dispersal of flightless weevils

**DOI:** 10.1002/ece3.7630

**Published:** 2021-05-03

**Authors:** Si‐Min Lin, Tsui‐Wen Li, Chia‐Hsin Liou, Ace Kevin S. Amarga, Analyn Cabras, Hui‐Yun Tseng

**Affiliations:** ^1^ School of Life Science National Taiwan Normal University Taipei Taiwan; ^2^ Biodiversity Program Taiwan International Graduate Program Academia Sinica Taipei Taiwan; ^3^ Department of Biology National Museum of Natural Science Taichung Taiwan; ^4^ Coleoptera Research Center University of Mindanao Davao City Philippines; ^5^ Department of Entomology National Taiwan University Taipei Taiwan

**Keywords:** avian‐mediated dispersal, colonization, endozoochory, frugivorous birds, migration, *Pachyrhynchus*

## Abstract

How flightless animals disperse to remote oceanic islands is a key unresolved question in biogeography. The flightless *Pachyrhynchus* weevils represent repetitive colonization history in West Pacific islands, which attracted our interests about how some weevils have successfully dispersed in the reverse direction against the sea current. Here, we propose endozoochory as a possible mechanism that the eggs of the weevils might be carried by embedded in the fruits as the food of frugivorous birds. In this study, *Pachyrhynchus* eggs were embedded in small pieces of persimmon fruits (*Diospyros kaki*) and fed to captive frugivorous birds. After digestion, 83%–100% eggs were retrieved from the feces of a bulbul (*Hypsipetes leucocephalus*) and two thrushes (*Turdus chrysolaus*). The retrieved eggs had hatching rates higher than 84%, which were not different from the control. In contrast, no egg was retrieved from the feces of the frugivorous pigeon (*Treron sieboldii*), which took a longer retention time in the guts. Our study identified that the eggs of *Pachyrhynchus* weevils are possible to be transported by internal digesting in some bird species.

## Graphical abstract

How flightless weevils disperse to remote oceanic islands is a fantastic issue in biogeography. Our study identified that the eggs of the weevils are possible to be transported by internal digesting in some frugivorous birds.

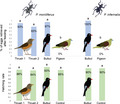

## 1 INTRODUCTION

Disjunctive distribution, meaning that closely related siblings or populations of a species to distribute across a large geographic barrier, is one of the major questions in biogeography. Oversea dispersal usually plays a key role in this process, which leads to colonization, population expansion, gene flow, and subsequent speciation events across the ocean (Claramunt et al., 2012; Smith et al., 2014). Nevertheless, a large proportion of such studies relies on current distributional patterns. Since colonization process is a comparatively rare event, direct experiments related to transoceanic dispersal abilities were scarce.

Dispersal ability decides the potential to colonize a new island. For volant species such as birds and butterflies, high dispersal ability facilitates movement among remote islands (Gillespie et al., 2012; Trierweiler et al., 2014). In contrast, flightless species are limited by their transoceanic dispersal ability (Gillespie et al., 2012). Various strategies have been reported from different flightless species. For example, the Tsudai stick insect (*Megacrania tsudai*) disperses through eggs across seawater probably because their eggshell contains sponge‐like structure aiding in buoyancy (Kobayashi et al., 2014). Aquatic invertebrates such as nematodes, rotifers, ostracods, copepods, tipulids, chironomids, and hemipterans might disperse by attaching on the plumage or feet of waterbirds (Frisch et al., 2007). Recently, a novel but important mechanism of “internal transportation” within the guts of birds was discovered. This mechanism, also known as endozoochory, has been proposed to transport a variety of invertebrates, such as zooplankton (Frisch et al., 2007), leaf beetle (Laux & Koelsch, 2014), chironomid larva (Green & Sánchez, 2006), Eurytoma seed wasps (Xiao, 2019), and even vertebrates (e.g., Killifish eggs (Silva et al., 2019) and cyprinid eggs (Lovas‐Kiss et al., 2020)).

*Pachyrhynchus* weevils (Coleoptera: Curculionidae) are a group of flightless insects distributed on islands ranging from southern Ryukyus to western New Guinea. With limited dispersal ability, these weevils form extremely high endemism on small oceanic islands with the highest diversity in the Philippines (Schultze, 1923; Yoshitake, 2017). However, some of the species have been proved to show long‐distance dispersal during the speciation process (Tseng et al., 2018). These genetic evidences contradicted our previous knowledge that some of these islands (e.g., Batanes Islands, Babuyan Islands, or Ludao and Lanyu of southeastern Taiwan) have never been connected to any others even in glacial periods because they are surrounded by very deep sea (Voris, 2000). Among the species which have been well documented for their spatial genetic pattern, *P. jitanasaius* Chen & Lin (2017) might be the first to be tested for their oversea dispersal ability, which could cross the ocean by rafting on floating fruit in the stage of eggs and larvae (Yeh et al., 2018). The salt tolerance of this species explains the historical gene flow among neighboring islands (Yeh et al., 2018). However, this experiment could not explain some events which dispersed in the reverse direction from the sea current across this region (Tseng et al., 2018).

Members of the *Pachyrhynchus obifer* species complex, referring to a diverse lineage comprising *P. obifer*, *P. infernalis*, *P*. *moniliferus,* and other congeners, represent a paradoxical case which has occurred long‐distance dispersal among West Pacific islands in both northward and southward directions (Tseng et al., 2018). A large proportion of adult *P*. *obifer* complex inhabit *Bischofia javanica* (Phyllanthaceae) and lay the eggs on a variety of parts of the tree including the fruits (personal observation). In the meanwhile, the fruits of *B*. *javanica* is a preferred food item for frugivorous birds, who help the seeds to disperse over long distance (Kawakami et al., 2009). Therefore, we hypothesized that frugivorous birds are potential vectors to transport the weevils across the ocean.

**FIGURE 1:**
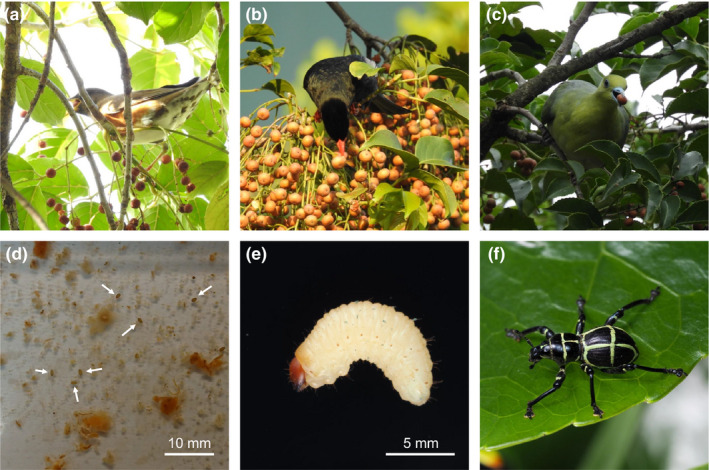
The fruits of *Bischofia javanica* are one of the major food items of frugivorous birds in West Pacific islands, and this plant is also the major host of some *Pachyrhynchus* weevils. The above three photos showed the fruits consumed by the three birds used in this study: the Brown‐headed Thrush (*Turdus chrysolaus*) (a), the Black Bulbul (*Hypsipetes leucocephalus*) (b), and the White‐bellied Green Pigeon (*Treron sieboldii*) (c). The below three photographs demonstrate the eggs retrieved from the feces (d), a larva (e), and an adult (f) of *P. moniliferus*. Photographed by Diting Li (a, b), Chi‐Hsuan Shao (c), Tsui‐Wen Li (d), and Bin‐Hong Ho (e, f)

In West Pacific islands, thrushes (Turdinae), bulbuls (Pycnonotidae), and pigeons (Columbidae) constitute the major members of the frugivorous guild. All the three taxa are often observed to feed on the fruits of *B. javanica* (Figure 1). The Brown‐headed Thrush (*Turdus chrysolaus*) is an abundant migratory bird wintering on the islands of Taiwan and the Philippines (Figure 1a). The Brown‐eared Bulbul (*Hypsipetes amaurotis*) and the Formosan Green Pigeon (*Treron formosae*) are distributed from southern Ryukyus, Green Island, Orchid Island, and the Philippines which form a congruent distribution pattern with the weevils. However, these two birds are not easily available because they distribute only on remote islets (Allen, 2020; Brazil, 2009). Therefore, we used their closely related taxa, the Black Bulbul (*H. leucocephalus*, Figure 1b) and the White‐bellied Green Pigeon (*T. sieboldii*, Figure 1c), as substitutions for the former two birds. These two birds also utilize frugivorous niche which is identical to their insular congeners; they feed frequently on fruits including *B. javanica*; and they are easily available in wildlife rescue systems or pet trades. We compared the survival rate of the eggs between the control group and gut‐passing group among different bird vectors and evaluate the possibility of oversea dispersal mediated by frugivorous birds.

## 2 METHODS

### 2.1 Study species

*Pachyrhynchus moniliferus* and *P*. *infernalis* were used to test whether the eggs can survive through birds' guts. The eggs used in this study were laid by captive‐bred populations established in the laboratory.

Two individuals of Brown‐headed Thrush were bought from a pet shop who provides wild bird for religious ceremonies, and both were released to the wild after the feeding experiment. A Black Bulbul was raised from a rescued fledgling in the campus of National Taiwan Normal University, and a captive‐bred White‐bellied Green Pigeon was acquired from a local pigeon farm. The latter two individuals were kept in a long‐term shelter before and after the experiment. All the procedures used in this study followed protocols approved by Institutional Animal Care and Use Committee (IACUC) of National Taiwan Normal University (license No. 109023).

### 2.2 Experimental procedures

In order to standardize the experiment, all the eggs we used were laid 3 days before the experiment. On the early morning of the experiment date, the bird was moved to a new and clean cage (60 × 45 × 45 cm). Five small pieces (6 × 6 × 6 mm each) of persimmon fruit (*Diospyros kaki*), embedded with a total of 30 eggs (6 eggs in each piece), were provided to the birds as their first meal in the morning. The weevil eggs are oval‐shaped with an average size of 1.0 × 0.5 mm (Figure 1d). They could be embedded 2 mm depth into the fruit by using tweezers. In this experiment, we used persimmon fruit to replace *B. javanica* fruit based on two reasons: (a) captive birds usually showed less willingness to consume *B. javanica* fruit compared to the colorful persimmon fruit; and (b) the orange color of the latter helped us to locate the weevil eggs from the feces.

From our pretests, bulbuls and thrushes defecated the persimmon fruit from 20 min to 1 hr, and the pigeon defecated 2 to 4 hr after feeding. Therefore, we collected all the feces of the thrushes and the bulbul during the first 2 hr and that of the pigeon during the first 6 hr after feeding. Feces were collected from a clean plastic tray under the cage and dissolved with water to retrieve the eggs (Figure 1d). The eggs were then transferred in a 50‐ml plastic bottle in a growth chamber with 12:12‐hr (L:D) photoperiod and 25°C, 70% relative humidity (RH). The eggs were checked everyday until the larvae were hatched. The larvae were fed on fresh sweet potatoes (replaced every week) until they become adults following protocols provided in Huang et al. (2018).

In the meanwhile, we randomly took 20 eggs without being digested by the birds and kept them in the growth chamber under the same condition. Hatching rates were compared between the experiment and the control groups.

### 2.3 Statistical analysis

The null hypothesis is that the eggs retrieved from the feces are similar among bird species. We used chi‐square tests with Yates correction to test the hypothesis, where Yates correction was used for small samples. We also compared the hatching rates of eggs between control and those through the gut of birds. The data were analyzed using R 3.6.3 software (R Core Team, 2020).

## 3 RESULTS

**FIGURE 2:**
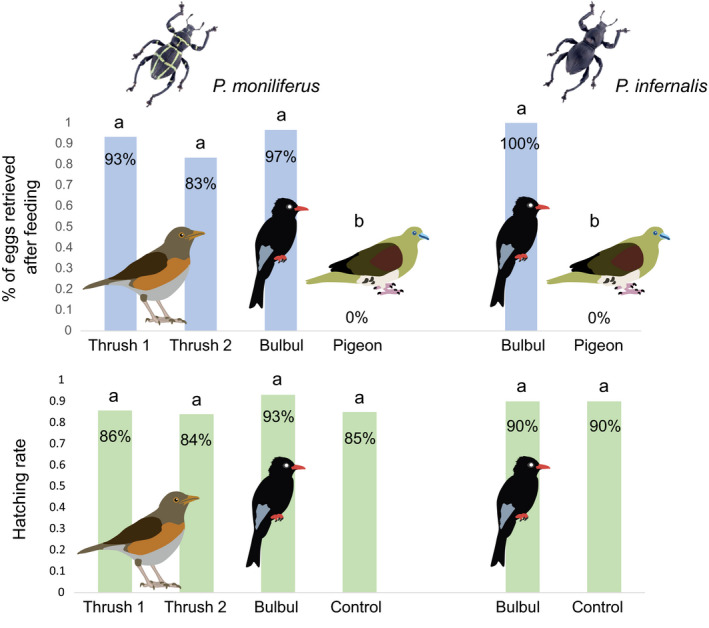
Number of eggs retrieved from the feces and hatching rate of the two *Pachyrhynchus* weevils passing through the gut of two Brown‐headed Thrush (*Turdus chrysolaus*), a Black Bulbul (*Hypsipetes leucocephalus*), and a White‐bellied Pigeon (*Treron sieboldii*). The birds are illustrated by Yun Ho

The time for persimmon fruit to pass through the gut of thrush and bulbul takes 20 min to 1 hr, and it takes almost 2 to 4 hr to pass through the gut of the pigeon. A total of 28 (93.3%), 25 (83.3%), and 29 (96.7%) eggs of *P. moniliferus* were retrieved from the feces of the two thrushes and the bulbul, respectively. However, no egg was found in the feces of the pigeon (Figure 2). Number of eggs retrieved was similar between the thrush and the bulbul, but was significantly different from that of the pigeon (*x* ^2^ = 40.357, *df* = 2, *p* < .001).

Since no egg was found from the feces of the pigeon, hatching rates of *P. moniliferus* were compared among the thrush, the bulbul, and the control. In the control group, 17 of 20 eggs (85.0%) were hatched in the growth chamber. In the experiment group, 24 (85.7%), 21 (84.0%), and 27 (93.1%) of the 30 eggs were hatched within 10 days (the two thrushes and the bulbul, respectively) (Figure 2). The hatching rates of retrieved eggs were not significantly different among the two birds and the control. About 10% hatchlings were raised to adults (Figure 1e,f) for each treatment group, which showed no difference from the control.

A similar result was observed from the eggs of *P. infernalis*, although only the bulbul and the pigeon were applied to this weevil. In this case, no egg was found in the feces of the pigeon and all the 30 eggs (100%) were retrieved from the feces of the bulbul. Twenty‐seven eggs hatched from the experimental group and 18 eggs hatched from the control, both yielded to a hatch rate of 90.0% (Figure 2). Also, similar to *P. moniliferis*, the larvae of *P. infernalis* were successfully raised to adults with similar ratio between the treatment groups and the control.

## 4 DISCUSSION

This study provided a potential dispersal mechanism of *Pachyrhynchus* weevils: eggs of weevils are possible to survive through the transport in the gut of frugivorous birds. Geographically, the biodiversity hotspot of *Pachyrhynchus* overlaps with the East Asian–Australasian Flyway, a major route of migratory birds passing through Ryukyu Islands, Taiwan, and the Philippines (Yong et al., 2015). Considering the huge amount of migrators including frugivorous species (e.g., the Brown‐headed Thrush used in the study and also other *Turdus* spp.), bird‐mediated transport is a possible explanation for oversea dispersal of the weevils.

In our study, the eggs of *Pachyrhynchus* weevils have a high survival rate after ingested by the Brown‐headed Thrush and Black Bulbul, but not for those digested by the White‐bellied Green Pigeon. Many factors may affect the egg survival rate, in which gut retention time and digestive system may play crucial roles. Gut retention time of seeds varies from ca. 20 to 170 min in passerines (Prunellidae, Muscicapidae, Aegithalidae, Paridae, and Fringillidae) (Herrera, 1984) to over 24 hr (maximum of 72 hr) in some mallard (*Anas platyrhynchos*) (Charalambidou et al., 2005). A long retention time in the gut has been proved either to enhance or to reduce the germination of seeds in some cases (Traveset, 1998). Nevertheless, we suspect that the effects to the eggs might be different from the seeds because the eggshell was directly facing the digestion process. Pigeons and their relatives have gizzards, which may increase the digestive efficiency by physically destroying the outlayer of the seeds/eggs (Lambert, 1989). Therefore, we deduce that the longer retention time might provide only negative effect to the eggs. In contrast, the hatching rates of the eggs passing through the guts of the thrush and the bulbul are comparable to the control group, indicating that these birds did not reduce the hatching rate of the weevils.

In addition to the hatch rate, the transportation efficiency of the birds also plays a crucial role. Flight speed of birds has been well established from major avian clades by using a variety of methods, such as tracking radar or wind tunnel experiments (Bruderer & Boldt, 2001; Alerstam et al., 2007; Hall & Heesy, 2011). From these literatures, the flight speed of migrating thrushes (*Turdus* spp.) was estimated to range between 11.4 and 12.5 m/s (41–45 km/h). The flight speed of bulbuls has never been estimated; nevertheless, as a medium‐sized passerine, the flight speed of bulbuls might be similar to the thrushes. Considering the geographic distance among West Pacific island chains (Appendix S1), migration among most these islands spends less than 1 hr for the passerines. We also noticed that the time required to reach some remote islands may take more than 2 hr, which is longer than the time for the eggs to stay in the gut. Nevertheless, the retention time in nature environment might be longer than the records in laboratory for the following reasons. First, the birds were fed only by the persimmon fruits for the first meal of the day. The retention time in the wild could be longer if the bird has already consumed other food items in the stomach. Second, the ripe persimmon fruits should be digested faster and easier than natural food items, such as the obviously harder *B. javanica* fruit.

In this experiment, we regret that *B. javanica* fruit was not an ideal bait to conduct the experiments because it was far less palatable than the persimmon fruit. Therefore, our experiment could not directly prove the correlation between *B. javanica* and the dispersal events. However, this plant was regarded as a new colonizer outside its original distribution through frugivorous birds such as the Brown‐eared Bulbul (Kawakami et al., 2009; Tanaka et al., 2010). Furthermore, it is one of the major host plants of this weevil complex. We conclude that even if the events might be uncommon, weevil eggs carried by the birds across the sea are a possible explanation for their oversea dispersal.

## CONFLICT OF INTEREST

The authors declare no conflict of interests.

## AUTHOR CONTRIBUTIONS

**Si‐Min Lin:** Data curation (equal); Resources (equal); writing‐original draft (equal). **Tsui‐Wen Li:** Conceptualization (equal); data curation (equal). **Chia‐Hsin Liou:** Conceptualization (equal); data curation (equal). **Ace Kevin** **S**. **Amarga:** Conceptualization (equal); resources (lead); writing‐review & editing (equal). **Analyn Cabras:** Conceptualization (equal); data curation (equal); resources (equal). **Hui‐Yun Tseng:** Conceptualization (equal); resources (equal); supervision (equal); writing‐review & editing (equal).

## ETHICAL APPROVAL

All the procedures used in this study followed protocols approved by Institutional Animal Care and Use Committee (IACUC) of National Taiwan Normal University (license no. 109023).

## Supporting information

Appendix S1

## Data Availability

All the data used in this study will be available in Dryad (https://doi.org/10.5061/dryad.6q573n5zj).
