# Supplementary figures and images for: Eggs survive through avian guts—A possible mechanism for transoceanic dispersal of flightless weevils
Source: Ecol Evol. 2021 May 3;11(12):7132–7. doi: 10.1002/ece3.7630 (PMC8216937; doi:10.1002/ece3.7630)

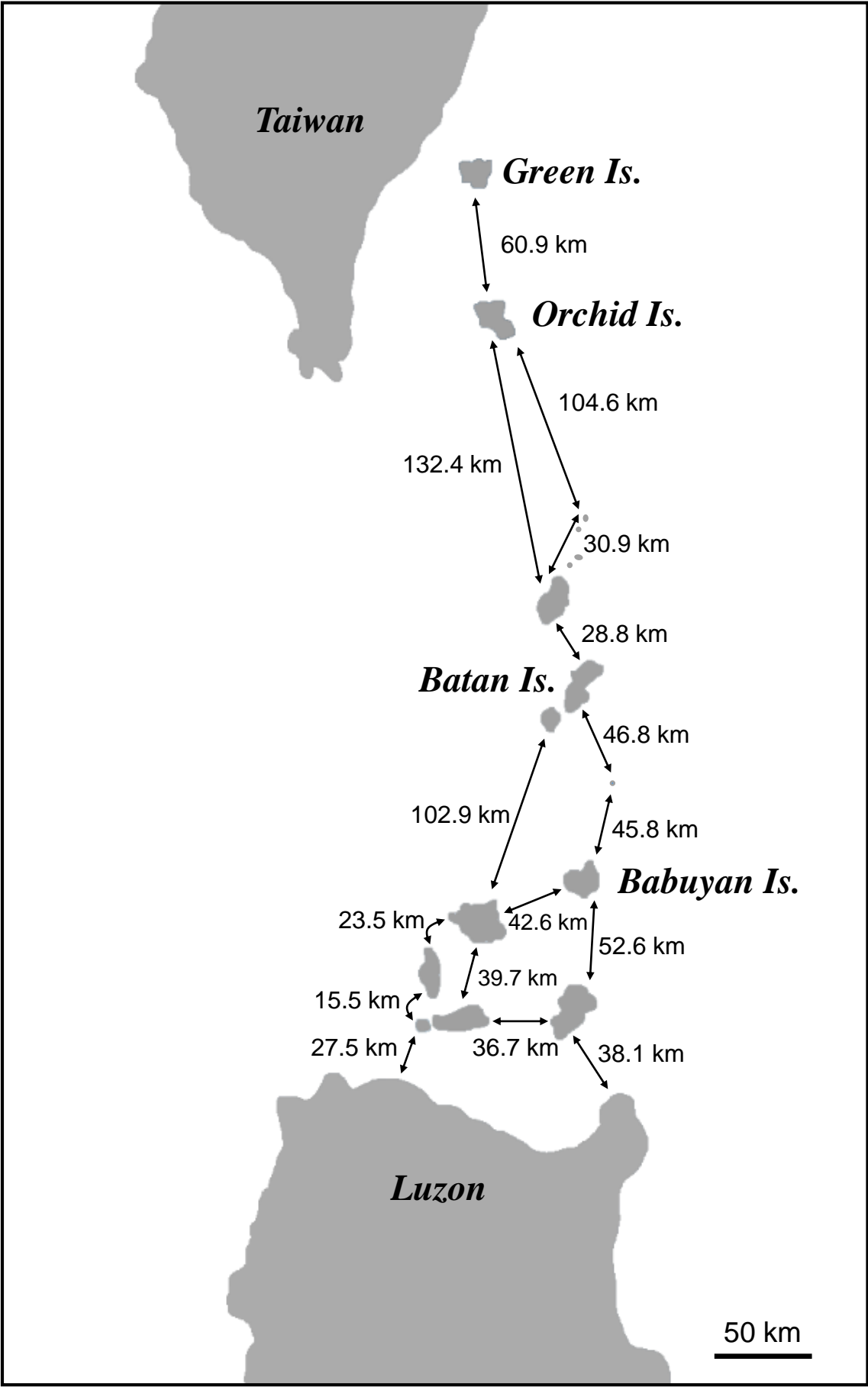

Supplement: Supplementary file 1 — Appendix S1 [file ECE3-11-7132-s001.pdf]
